# Reversal of phenotypes of cellular senescence by pan-mTOR inhibition

**DOI:** 10.18632/aging.100872

**Published:** 2016-02-05

**Authors:** Hannah E. Walters, Sylwia Deneka-Hannemann, Lynne S. Cox

**Affiliations:** ^1^ Department of Biochemistry, University of Oxford, South Parks Road, Oxford, OX1 3QU, United Kingdom; ^2^ Oxford BioMedica Plc, Oxford, OX4 6LT, United Kingdom

**Keywords:** cellular senescence, aging, mTORC1, mTORC2, rapamycin, AZD8055

## Abstract

Cellular senescence, a state of essentially irreversible proliferation arrest, serves as a potent tumour suppressor mechanism. However, accumulation of senescent cells with chronological age is likely to contribute to loss of tissue and organ function and organismal aging. A crucial biochemical modulator of aging is mTOR; here, we have addressed the question of whether acute mTORC inhibition in near-senescent cells can modify phenotypes of senescence. We show that acute short term treatment of human skin fibroblasts with low dose ATP mimetic pan-mTORC inhibitor AZD8055 leads to reversal of many phenotypes that develop as cells near replicative senescence, including reduction in cell size and granularity, loss of SA-β-gal staining and reacquisition of fibroblastic spindle morphology. AZD8055 treatment also induced rearrangement of the actin cytoskeleton, providing a possible mechanism of action for the observed rejuvenation. Importantly, short-term drug exposure had no detrimental effects on cell proliferation control across the life-course of the fibroblasts. Our findings suggest that combined inhibition of both mTORC1 and mTORC2 may provide a promising strategy to reverse the development of senescence-associated features in near-senescent cells.

## INTRODUCTION

Cellular senescence is a hallmark of aging [1] and senescent cells accumulate with age *in vivo* in mammals [2, 3]; this is thought to drive aging by limiting tissue replicative capacity and causing tissue dysfunction (reviewed in [4]). Senescent cells can be characterized by significant alterations in phenotype: they exhibit a large, flat, vacuolated and granular morphology with accumulation of lipid droplets and visible stress fibers, together with increased lysosomal content [5]. Proliferative arrest accompanies senescence, shown by down-regulation of proliferative markers such as Ki67 as well as increased expression of mediators of senescence, such as the cyclin kinase inhibitors p16^INK4A^ and p21^CDKN1^ [6-9]. Deletion of the p21 gene can prolong lifespan in telomerase-null mice [10] and clearance of p16-expressing senescent cells *in vivo* can rejuvenate aged mice [11]. Moreover, telomerase reactivation suppresses premature aging phenotypes in telomerase knock-out mice [12-14]. Taken together these key findings strongly support the argument that senescent cells are detrimental in older animals. Developing strategies to delay the onset of senescence or remove senescent cells therefore may provide a route to preventing age-related disease.

Targeting senescence as a means to combat aging and age-related diseases is, however, challenging due to its antagonistically pleiotropic nature – any treatment needs to limit the deleterious impacts of senescent cells without impacting the potent barrier against tumorigenesis. While caloric restriction has been reported to extend healthspan in macaques [15], the most promising candidate for a longevity therapeutic in mammals is rapamycin [16]; (reviewed [17, 18]). Rapamycin is a macrolide antibiotic produced by *Streptomyces hygroscopius*, discovered in the soil of Easter Island [19]. It is clinically licensed for immunosuppression in kidney transplant patients and for renal cell carcinoma treatment due to its broad inhibitory effects on cell growth and proliferation [20]. As discovered through *S. cerevisiae* genetic screens [21], rapamycin mechanistically acts by binding the protein FKBP12, producing a complex which can bind and inhibit mTOR, a conserved eukaryotic Ser/Thr kinase. mTOR constitutes the point at which diverse environmental signals are coordinated into a cellular response, regulating pathways including cell growth, proliferation, survival, motility and protein synthesis [22-24]. mTOR is present in two complexes in metazoa, mTORC1 and mTORC2, which have different components and functions [22]. Rapamycin inhibits mTORC1, but chronic treatment may also disrupt mTORC2. Rapamycin does not inhibit the phosphorylation of all mTORC1 substrates equally: it completely inhibits phosphorylation of S6K1 while only partially blocking the phosphorylation of 4EBP1 [25]. A crystal structure of mTOR, rapamycin and FKBP12 [26] suggests that this may be due to differential substrate access to the kinase active site; this is supported by further crystallography data [27]. While rapamycin extends lifespan in mice even when administered in middle age [16], it has significant side-effects that may limit its use in humans. We have therefore explored the potential of second generation rapalogs i.e. pharmacological agents that inhibit mTORC but act not through binding to FKBP12 but instead as mTORC-specific ATP mimetics [28]. AZD8055 is an ATP-competitive inhibitor of mTOR kinase in both mTORC1 and mTORC2, with an IC_50_ of 0.8 nmol/L, with ∼1000-fold selectivity for mTOR over other PI3K family members and no significant activity against a large panel of other cellular kinases [29]. AZD8055 has anti-proliferative effects similar to those of rapamycin and has been taken forward into clinical trials against various forms of cancer [30].

To date, studies examining the impact of rapalogs on aging has required chronic drug administration (e.g. [16]), an approach that may not be acceptable for prophylactic avoidance of age-related disease in the general human population. Here, we test whether acute mTORC inhibition can alter features of senescence in cells that have already undergone a large number of population doublings (PD) – as they are about to undergo senescence but are currently still proliferating, we term these populations ‘near-senescent’. Such high cumulative PD (CPD) near-senescent cells show many signs characteristic of senescence including increased size and granularity, SA-β-gal staining, high lysosomal content and accumulation of actin stress fibers. They are still capable of cell proliferation, albeit with a reduced rate of proliferation compared with cells at lower CPD. Here, we test the effect of inhibiting both mTORC1 and mTORC2 using the TOR-specific ATP mimetic AZD8055. Remarkably, we demonstrate significant reversal of major phenotypes of senescence on short term low dose pan-TOR inhibition. We therefore suggest that AZD8055 may prove useful in modulating health outcomes in late life.

## RESULTS

### Morphological rejuvenation of near-senescent cells

**Figure 1:**
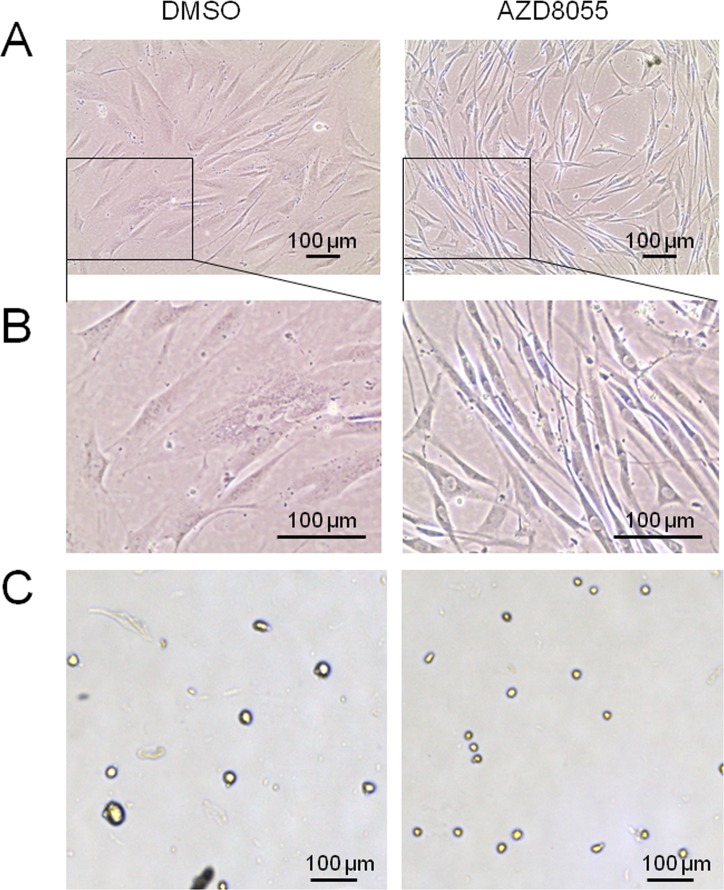
AZD8055 causes major changes in near-senescent fibroblast morphology and size HF043 fibroblasts at high CPD were incubated with AZD8055 or equivalent volume of DMSO for 7 days. (**A**) Cells were observed *in situ* by phase contrast microscopy. (**B**) Magnified images from (**A**). (**C**) Cells were harvested by trypsinization and cell suspension analysed using a Cellometer T4; images were obtained at x20 magnification.

We first set out to test the impact of AZD8055 treatment on cell morphology, as this represents a very useful biomarker of cellular senescence in fibroblasts [31]. Near-senescent diploid human neonatal foreskin fibroblasts (HF043) were obtained by serial passaging in the absence of any drug treatment until they started to show signs characteristic of early senescence (such as enlarged cell size, decreased proliferation rate and elevated p21 levels – see supplementary Fig 1). Cell populations showing senescent-like phenotypes were harvested and seeded into parallel culture flasks/plates and then treated with AZD8055 (or DMSO vehicle control) for 7 days. Drug doses were chosen to mimic serum concentrations of animals treated with rapalogs (e.g. [16]); these doses were found to have little or no toxicity, though they did slow down the rate of cell proliferation (data not shown). Overall morphology was observed by phase contrast microscopy. As expected, control cells demonstrated increased size, vacuolization and granularity typical of cells as they approach senescence (DMSO, Fig 1A, B). By contrast, one week's treatment with 35 nM AZD8055 led to marked alteration in morphology, with cells re-acquiring the classical spindle morphology characteristic of low CPD proliferating fibroblasts (Fig 1A, B). This result has been verified in several other populations of high CPD HF043 cells, including cells at CPD 88 (note that this fibroblast line HF043 reaches replicative senescence at ∼CPD 95).

To determine whether these apparent cell size changes are quantifiably different following AZD8055 treatment, we harvested cells by gentle trypsinization so that cells detach from the substrate and round up to ovoid/spherical shapes (Fig 1C). Cell diameters in these harvested populations were measured using a Cellometer T4 (see Methods). Low CPD cells (CPD 36) showed a slight though not significant effect of AZD8055 in decreasing diameter (Table 1). However, consistent with the changes observed by phase contrast microscopy (Fig 1C), we found a highly significant reduction in cell diameter of cells at CPD 73 from a mean of 27.7 μm (DMSO control) to 24.7 μm on AZD8055 treatment (unpaired t-test, p<0.02, Table 1), a cell diameter highly similar to that of low CPD untreated cells. Again, this was replicated in several other populations of near-senescent HF043 cells, producing highly similar results.

**Table 1:** Mean cell diameter on AZD8055 treatment

	DMSO	AZD8055	
**CPD 36**	25.7 (148)	24.5 (49)	NS
**CPD 73**	27.7 (120)	24.7 (64)	*
	*	NS	

Cells were grown with and without 7 day exposure to 70 nM AZD8055 compared with DMSO control at CPD 36 (low CPD) or CPD 73 (near-senescent). Unpaired t tests were used to assess significance (>40 cells analyzed per sample). NS = not significant. n values for number of cells measured are given in parentheses below each diameter value. * denotes significant at p<0.02.

### Loss of mitotracker signal on AZD8055 treatment

**Figure 2:**
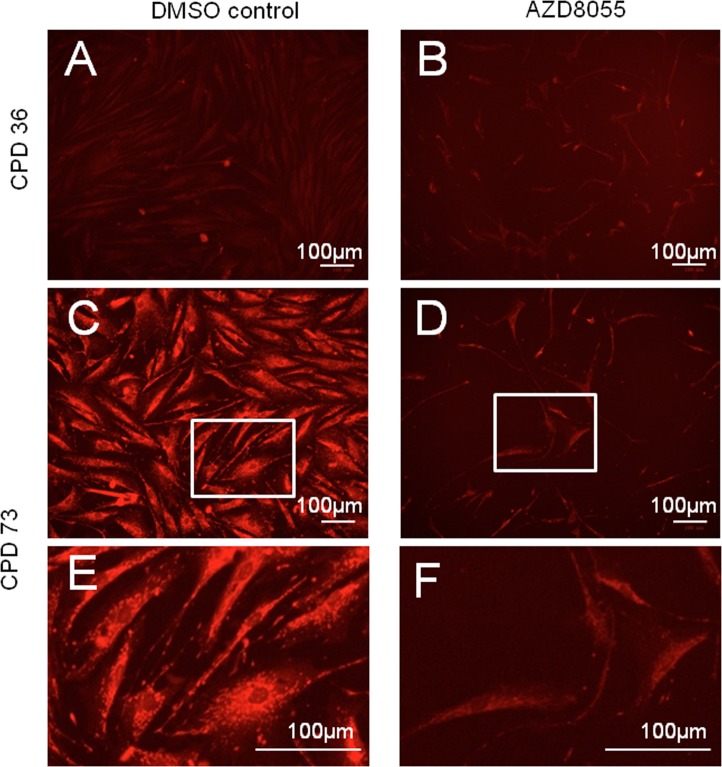
Decreased senescence-related mitochondrial signal on AZD8055 treatment Fibroblasts were incubated with DMSO (**A, C, E**) or AZD8055 (B, D, F) for 7 days prior to staining with MitoTracker Red and imaging with a BioRad Zoe fluorescent imager. (**A, B**) show cells at early stages of culture (CPD 36), while C-F show cells at CPD 73 at time of drug treatment. (**E, F**) are magnified from **C, D** (respectively, region magnified shown by white box). Scale bar 100 μm. Note that gain and exposure time were the same in all photomicrographs.

Mitochondrial biomass increases as cells approach senescence [32] possibly as a compensatory mechanism for increasingly inefficient mitochondrial activity. We therefore used a mitochondrial-specific probe, Mitotracker Red, to label mitochondria in low and high CPD cells with acute mTORC inhibition. Mitochondria were detected as reticular networks throughout the fibroblasts, though at low population doublings the signal was relatively weak both without (Fig 2A) and with (Fig 2B) AZD8055 exposure. By contrast, a high Mitotracker signal was detected in near-senescent cells at CPD 73 (Fig 2C, E), consistent with increased ROS in the mitochondria of aged cells. This signal was dramatically reduced on AZD8055 treatment (Fig 2D, F), to levels similar to those detected in cells early in their proliferative lifespan (ie at low CPD (Fig 2A, B)).

### Effects of AZD8055 on lysosomes and SA-β-galactosidase

**Figure 3:**
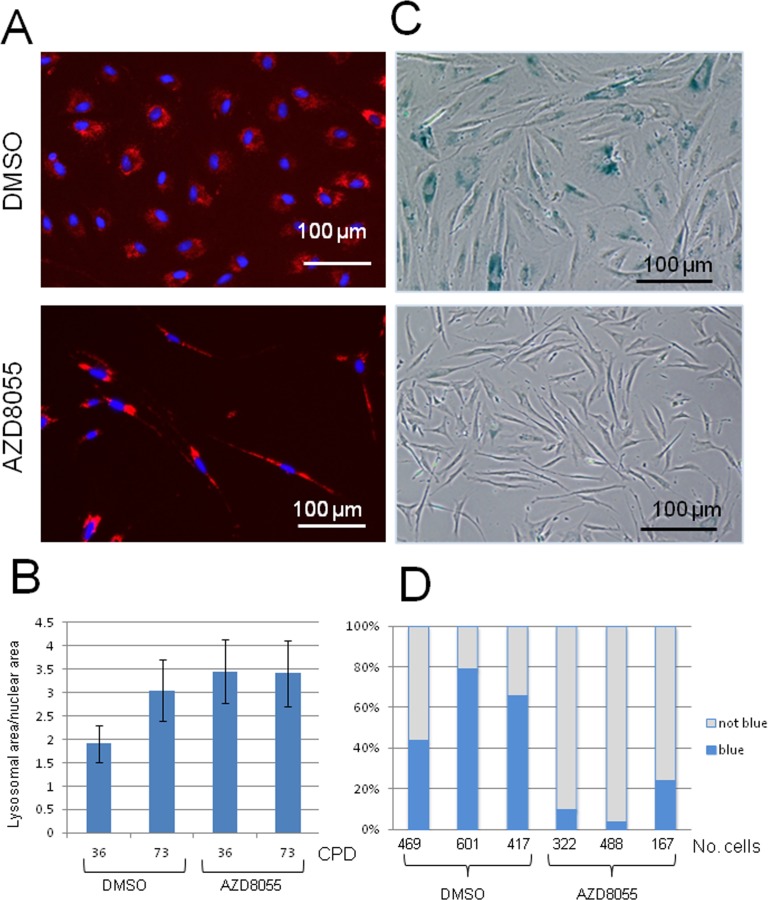
Lysosomal distribution and function are altered on AZD8055 treatment Fibroblasts at CPD 73 were treated with AZD8055 or DMSO for 7 days then imaged for (**A**) lysosomes using LysoTracker Red. (**B**) Quantification of lysosomal signal over nuclear signal was conducted using ImageJ analysis, for cells at early CPD (36) and those nearing senescence (CPD73) with either AZD8055 treatment at 35 nM or DMSO control. (**C**) SA-β-gal staining of fibroblasts following one week of AZD8055 treatment. (**D**) Three independent biological replicates were each assayed by manually scoring SA-β-gal positive cells - numbers scored for each sample are shown under the graph. Difference between the means of the AZD8055-treated and DMSO controls are significant at p<0.005 using one tailed student t test.

The cellular lysosomal content increases gradually with biological age of cells and can be used as a biomarker of senescence [5]. To assess the effect of mTORC inhibition on lysosomal content, near-senescent fibroblasts were treated with AZD8055 or DMSO for 7 days and then lysosomes were labelled using Lysotracker Red. Notably, treatment of near-senescent cells with AZD8055 led to a marked redistribution of the lysosomal signal, from a diffuse perinuclear pattern seen in control cells to a more intense pattern distributed along the axis of the cell on drug treatment (Fig 3A), highlighting the overall shift in near-senescent cell shape on AZD8055 treatment to a more spindle-like morphology. As anticipated, lysosomal content increased as control cells reached late passage compared with early passage (assessed quantitatively using Image J, Fig 3B). The lysosomal signal was elevated in early passage cells treated with AZD8055 and this did not alter on cell ageing (Fig 3B).

In addition to increased lysosomal loading, senescent cells - as well as those nearing senescence [31] – also show a major shift in biochemical activity within lysosomes, reflected by the canonical marker SA-β-gal (senescence-associated-β-galactosidase) [5]. We there-fore assessed the percentage of near-senescent cells staining positive for SA-β-gal upon treatment (Fig 3C, D). On average, more than 56% of control cells at high CPD stain positive for SA-β-gal, while this was markedly reduced to only 12% after 7 days of AZD8055 treatment (Fig 3D). The difference is highly significant (p<0.005, one-tailed student t test, n= 3 independent biological replicates). This marker of senescence is therefore lost on AZD8055 treatment, suggesting a reversal of specific cellular metabolic features associated with senescence.

### Major rearrangement of the actin cytoskeleton accompanies reversal of other senescent biomarkers

**Figure 4:**
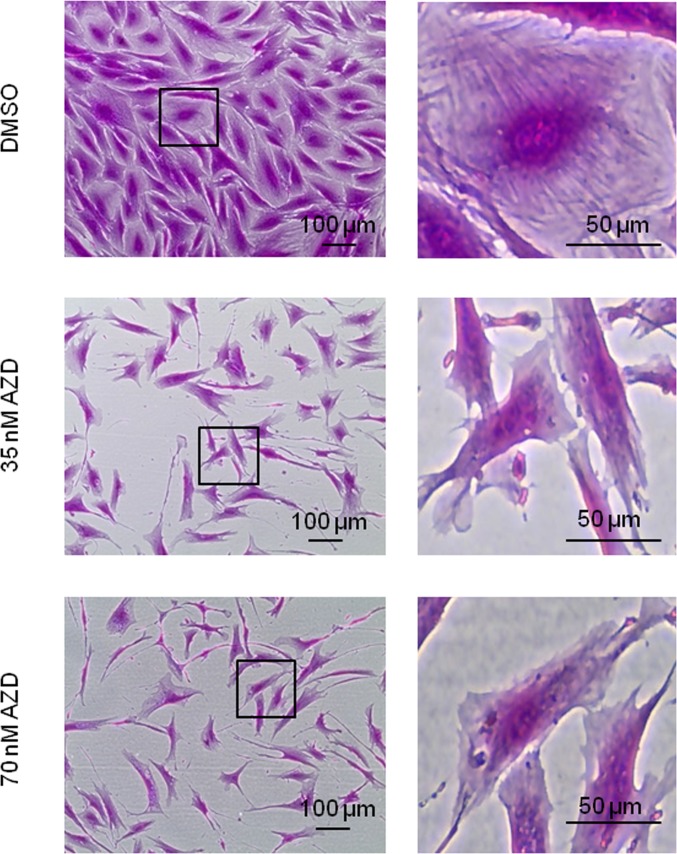
Loss of filamentous structures in AZD8055-treated cells Fibroblasts at CPD 73 exposed to 35 nM or 70 nM AZD8055 or equivalent volume of DMSO for 1 week were fixed and stained using sulforhodamine B, then imaged by phase contrast microscopy. Left panel imaged with X40 objective; right panel magnification of regions outlined by black boxes.

To further analyze these morphological differences, we stained near-senescent cells with sulforhodamine B with and without prior exposure to AZD8055 for 7 days; this dye is more usually utilized for cytotoxicity screening and biomass measurements [36] but we also find it very informative for microscopic analysis, by either phase contrast (Fig 4) or fluorescence microscopy (Ex480 Em520, not shown). At the molecular level, SRB binds basic amino acid residues under mild acidic conditions, thus staining the cellular protein content. The negative impact on cell proliferation of mTORC inhibition is obvious here as cell density after 7 days of drug exposure is much lower than in the control, even though identical cell numbers were seeded. As consistently observed, AZD8055 treatment again produced a notable decrease in cell size compared with control cells (Fig 4). Additionally, SRB staining highlights filamentous structures which are particularly visible in the control near-senescent cells (Fig 4, DMSO) and which disperse upon AZD8055 treatment (Fig 4, 35nM and 70 nM AZD8055).

**Figure 5:**
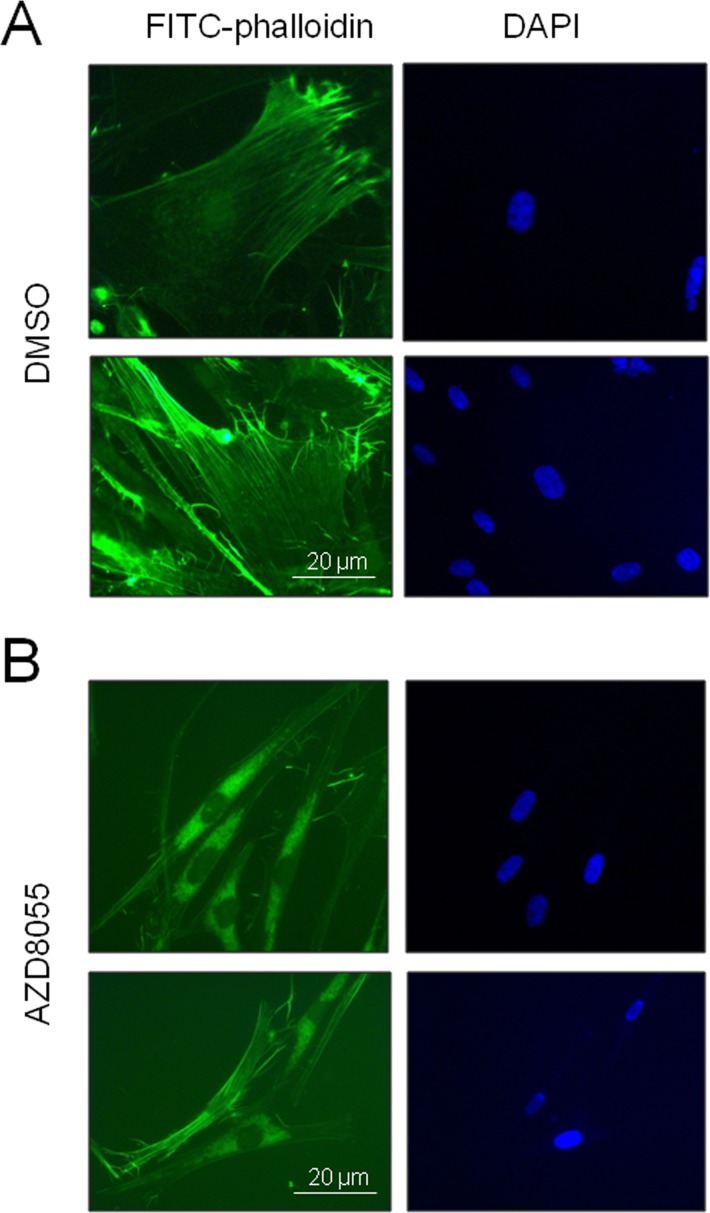
AZD8055 treatment reverses stress fiber formation in near-senescent cells Late CPD HF043 fibroblasts were exposed to (**A**) DMSO vehicle control or (**B**) AZD8055 for 7 days then fixed and stained for actin using FITC-phalloidin. Imaging was carried out using a Zeiss AxioSkop II with x100 oil immersion lens. Representative images are shown for each treatment condition.

To determine whether these filamentous structures observed by SRB staining were indeed actin stress fibers, we stained cells using the actin-specific fluorescent dye FITC-phalloidin. While stress fibers were clearly visible in control late passage fibroblasts (Fig 5A), after 7 days of AZD8055 treatment such fibers became much less prominent and actin instead appeared more centrally distributed (Fig 5B), suggestive of a shift from filamentous to globular conformation. Most notably, the cells changed morphology from enlarged amorphous cells to much smaller, thinner and spindle shapes characteristic of low CPD fibroblasts. Thus AZD8055 appears to trigger a rearrangement of the actin cytoskeleton in late passage cells to a state more usually seen in proliferating cells at low CPD.

### Protein changes on short term AZD8055 treatment

**Figure 6:**
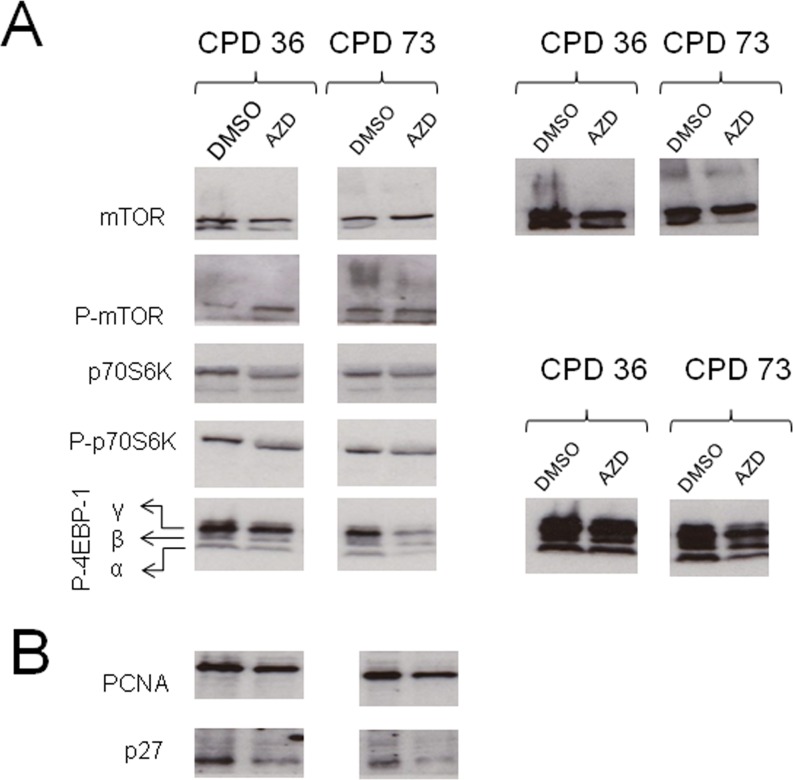
Alteration in protein levels and phosphorylation on short term AZD8055 treatment Cells at CPD 36 or CPD 73 were seeded into T25 flasks and grown in medium containing AZD8055 or DMSO for one week. After harvesting, cells were pelleted and lysed in RIPA with protease and phosphatase inhibitors, separated on SDS-PAGE then immunoblotted for the indicated proteins. (**A**) Components of the mTOR signaling pathway, (**B**) Cell cycle proteins. The panels on the right show longer exposure images of the same panels on the left, to highlight bands that are faint in the shorter exposure images.

The phenotypic and morphological changes we observe on AZD8055 treatment are highly suggestive of rejuvenation. We therefore asked whether such changes are accompanied by alterations in protein levels or modification, particularly addressing components of the mTOR signaling pathway (Fig 6A) and cell proliferation associated proteins (Fig 6B) by immunoblotting cell lysates from low CPD (CPD 36) or near-senescent (CPD 73) fibroblasts. We find that at low CPD, there is little apparent difference in total levels of mTOR, but phospho-mTOR increased upon AZD8055 treatment (Fig 6). The antibody used to detect total mTOR recognises a doublet in control cells; the lower band of the doublet is decreased in both low and high CPD drug-exposed cells. In near-senescent fibroblasts, an additional high molecular weight smear is detected both with anti-mTORC and anti-phospho-mTORC antibodies – this higher molecular weight form is also detected in DMSO control cells at low CPD but not on AZD8055 treatment.

The translation initiation inhibitor 4EBP-1 is a well-documented target of mTORC1 kinase. On Western blotting we observe a marked impact on phospho-rylation of translation initiation inhibitor 4EBP-1 with AZD8055 treatment, such that phosphovariants α and β [37] were detected at lower levels in cells on treatment with AZD8055, particularly for those near senescence (Fig 6A), – note that these differences are not due to uneven loading as they are strips taken from the same gel lane of the same membrane as the phospho-mTOR blots (and this finding was replicated over several experiments, not shown). We also find that levels of the replication clamp PCNA and the cell cycle regulator p27 are both diminished on AZD8055 treatment (Fig 6B).

### Release of drug treatment leads to normal senescence trajectory

**Figure 7:**
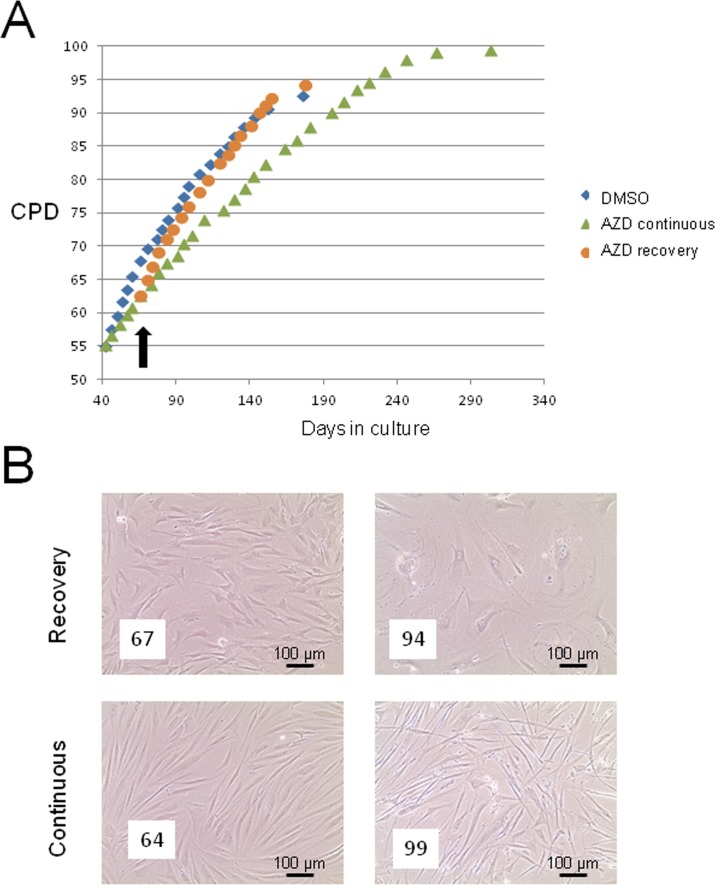
Short term AZD8055 exposure does not immortalise or transform primary human skin fibroblasts (**A**) HF043 fibroblasts at CDP55 were treated with 35nM AZD8055 either continuously (green triangles) or for 5 population doublings followed by release from drug (black arrow indicates time of drug removal; growth curve shown by orange circles) compared with cells incubated with DMSO as control (blue diamonds). The end point of the growth curve denotes when cells ceased proliferation and became fully senescent (NB cells under continuous AZD80055 treatment continued for several PDs beyond the graph shown but also eventually died). (**B**) Phase contrast photomicrographs of cells in culture either following release from AZD8055 (‘Recovery’) or under continuous drug treatment (‘Continuous’). The left panel shows cells 8 days after drug release and the right panel shows cells at very late CPD: CPDs are shown in white boxes within the micrographs.

Given the significant impact of AZD8055 in apparently reversing senescence phenotypes, it was important to address whether release from drug treatment might result in unanticipated changes in cell behaviour such as hyper-proliferation, immortalisation or even neoplastic change. We therefore treated cells at ‘middle age’ (CPD 55) with 35 nM AZD8055 and maintained drug treatment continuously or released cells from drug at CPD 60 (black arrow in Fig 7A). Cells were then grown under standard tissue culture conditions (see Methods) until replicative senescence. Proliferation rates rapidly returned to those of control cells on release from AZD8055 treatment (Fig 7A, compare blue diamonds with orange circles). Released cells reached senescence at almost the same cumulative population doubling as DMSO controls and showed no long term alteration in growth characteristics or morphology compared with DMSO controls. By contrast, continuous dosing of cells with AZD8055 did restrain proliferation (green triangles, Fig 7A), consistent with inhibition of mTORC1 and negative effects on translation, presumably through blocking inactivation of 4EBP-1 (see also Fig 6). This was further studied by observing cell morphology *in situ*. Shortly after the time of release from drug treatment (8 days in Fig 7B), there is little difference in cell size or shape between the ‘recovery’ population and those under continuous drug treatment. By contrast, at very late cumulative population doublings (CPD94), released cells showed a classic senescent morphology with greatly enlarged cell size and many cells were rounded in shape (Fig 7B, CPD94), while continuous AZD8055 exposure from mid-CPD resulted in maintenance of a spindle cell morphology characteristic of low CPD cells- notably, cells at extremely high population doublings (CPD99) that were continuously exposed long term to AZD8055 were very similar morphologically to the appearance of near-senescent cells after only one week of drug treatment (compare Fig 7B CPD 99 ‘Continuous’ with Fig 1B). From these findings, we conclude that brief exposure to AZD8055 does not have any long-lasting adverse effects on proliferative properties of skin fibroblasts, an important finding if AZD8055 is to proceed further into whole animal aging studies.

## DISCUSSION

In this paper, we report that short-term inhibition of mTORC1 and mTORC2 by administration of AZD8055 can reverse morphological and biochemical phenotypes of senescence in near-senescent human fibroblasts, including reduction in cell size and granularity, loss of SA-β-gal staining and reacquisition of fibroblastic spindle morphology. Previous use of mTORC1 inhibitors such as rapamycin has required continuous drug exposure to delay the onset of senescence and hence delay organismal aging (e.g. [16, 39]), so this represents a significant new means of altering senescence in primary cells. Cellular senescence provides an important barrier to tumorigenesis, but senescent cells indirectly contribute to a pro-neoplastic environment (‘good citizens, bad neighbors’ [38]). As a non-proliferating component of the tissue, senescent cells are also unable to contribute to tissue maintenance and repair. Hence pharmacologic strategies to relieve senescence are likely to have beneficial consequences for organismal health during aging.

mTORC inhibition provides a highly promising route to improving health span of older mammals. As well as increasing overall lifespan of mice [16], many measures of health are improved on administration of the mTORC1 inhibitor rapamycin [39]. Given the huge projected increase in age-related dementias with demographic change in Western populations [40], it is of particular importance that rapamycin increases blood brain flow, reduces amyloid tangles and plaques and improves cognitive function in mouse models of Alzheimer's disease [41-43]; it also shows promise in Parkinson's disease [44], other neurodegenerative disorders [45] and potentially age-related decline in cognitive function distinct from disease [46]. Far from its original role as an immunosuppressant (at high dose), rapamycin actually improves immune function in response to antigenic challenges when used at lower doses [47]. An orally available derivative, everolimus, shows good bioavailability and has shown promise in stimulating anti-viral immunity in older people on challenge with influenza vaccine [48], though the dose that improves immune memory may negatively impact on response to acute infection [49]. Furthermore, AZD8055 may be immunostimulatory [50].

Autophagy has been suggested as a potential mode of action by which mTORC1 inhibitors exert putative anti-aging effects, as mTORC1 phosphorylates and inactivates the autophagy-initiating kinase ULK1. Other approaches that stimulate autophagy can also improve health outcomes on aging, such as spermidine suppression of immunosenescence in mice [51]. While we investigated whether stimulation of autophagy could be responsible for the rejuvenated phenotypes seen upon AZD8055 treatment of late passage cells, our results are as yet inconclusive.

While rapamycin and similar molecules all act through binding to the FKBP12 protein - which then associates with the mTOR kinase and partially occludes the active site in mTORC1 [26], altering substrate recognition - second generation mTOR inhibitors have been designed instead to act as ATP mimetics that are highly specific for mTOR above other PI3K-family kinases and all other cellular kinases [29, 52]. Here, we find that administration of one such ATP mimetic, AZD8055, used at low concentration for as little as 7 days can markedly alter aging phenotypes in primary human skin fibroblasts, including redistribution of lysosomes and alteration in metabolism. Importantly, levels of senescence-associated β galactosidase, a marker of cellular senescence [5] drop in a highly significant manner, suggesting that lysosomal metabolism has shifted away from that characteristic of senescent cells.

Furthermore, we find a very robust reversal of morphological phenotypes of senescence, from enlarged, flattened, amorphous cells to an elongated spindle morphology characteristic of low CPD proliferating skin fibroblasts. The cell sizes we observe are entirely consistent with those previously reported for normal low CPD fibroblasts [57] and appear to be cell-intrinsic in both low CPD fibroblasts and higher CPD drug-treated cells, i.e. AZD8055 administration appears to reset the endogenous cell size regulation reported in [57]. In order to achieve this marked alteration in size and shape, a significant change in the organisation of the cytoskeleton must be taking place – we observe loss of filamentous structures (Fig 4) together with complete rearrangement of actin microfilaments from prominent stress fibers in control near-senescent cells to a central diffuse distribution more reminiscent of pools of globular actin (Fig 5). We suggest that this might be accompanied by an increase in cell motility as the AZD8055 treated cells show leading edge lamellae with some associated actin filaments (Figs 4 and 5) indicative of highly motile cells [57].

So how is AZD8055 causing such a major reorganisation of cellular structure? AZD8055 inhibits mTOR not only in mTORC1 but also when in complex with Rictor and PRR5 i.e. within mTORC2 [22]. Whilst rapamycin can lead to unwanted side effects such as decreased glucose homeostasis and increased diabetes risk [58] it is likely that this is caused by activation of a feedback loop with AKT on chronic (and high dose) rapamycin inhibition of mTORC1 [59] and Ying-Yang1 interaction [58] rather than via mTORC2 inhibition. Abrogation of mTORC2 activity leads to loss of phosphorylation of Rac/Cdc42 and downstream PAK; the consequence is relief of inhibition of cofilin, resulting in a shift in the equilibrium of actin from f-actin to g-actin [60]. Unlike the aberrant cell sizes caused by actin-disrupting drugs such as cytochalasin [57], we observe cell lengths typical of normal fibroblasts on AZD8055 treatment. Hence AZD8055 allows the cell to reorganise actin and restores the ability of cells to regulate size and shape. It has been suggested that f-actin polymerisation is critical for both short-term memory and long term potentiation in the brain [60]. This does raise the worrying prospect that long term AZD8055 administration may impact on cognitive function, though such fears associated with use of rapamycin have proven groundless; rapamycin actually improves learning and memory in older mice [46]. It remains to be seen whether AZD8055 is similarly beneficial to cognitive function.

Any intervention designed to tackle the problem of senescent cells during aging must not cause long-term harm, particularly neoplastic change. As such, it will be crucial to investigate whether AZD8055 treatment can reverse features of oncogene-induced senescence and senescence induced by other stresses such as DNA damage, as well as the replicative senescence investigated here. Other mooted anti-aging strategies such as telomerase reactivation carry huge cancer risk; indeed pre-neoplastic lesions become highly aggressive in telomerase-reactivated mice [61]. Drugs and potential nutraceuticals that activate sirtuins show promise in terms of improved tissue function [62]; however, the strong association of some of the sirtuin protein deacetylases with cancer [63] has to be carefully considered, especially since epigenetic shifts in senescent cells are associated with increased neoplastic transformation [64]. Other approaches include targeting the SASP (senescence-associated secretory phenotype), and recent evidence indicates that rapamycin may disrupt the SASP by preventing IL1A translation [70]. Furthermore, inhibition of stress signaling through p38 MAPK kinases looks promising in Werner syndrome progeroid models of aging [65] though the therapeutic window is extremely narrow, which may limit clinical use. Since AZD8055 is already in clinical trials as an anti-cancer therapeutic [30], we were optimistic that it may be less risky in terms of possible adverse effects in stimulating cell proliferation and/or neoplastic change. However, it was essential to test whether the drug had negative effects on cell cycle control.

The rate of proliferation of fibroblasts treated with AZD8055 is greatly diminished compared with controls. This is likely to be a consequence, at least in part, of AZD8055 inhibition of protein synthesis, as its inhibition of mTORC1 then blocks relief of translational inhibition by downstream target 4EBP-1. AZD8055 has been reported to alter the profile of translated mRNAs in the cell. In particular, a subset of mRNAs with a 52 terminal oligopyridine tract are affected [55], including a host of ribosomal protein mRNAs together with those encoding cell cycle factors such as the replicative helicases MCMs and the sliding clamp PCNA [56]. Hence factors needed both for increasing cellular mass through ribosomal biogenesis, and those directly involved in critical cell cycle processes such as DNA replication, are kept at lower levels in mTORC-inhibited cells. Consistent with this, we find significant alteration in phospho-4EBP-1 isoforms and decreased PCNA levels on AZD8055 treatment that correlate with the decreased growth rates of treated cells. At first sight, this finding is counterintuitive: a treatment that reverses features of senescence leads to decreased PCNA, but low PCNA levels are characteristic of cells undergoing cell cycle arrest and geroconversion to the senescent state [71, 72]. However, the important point is that the AZD8055 treated cells (unlike senescent cells) have not lost proliferative potential - on release from drug treatment, they regain normal proliferative capacity. While rapamycin treatment of cultured rat cells (that do not show telomere-dependent senescence) has been reported to switch cells to a non-senescing state [66], we believe it is critically important that the short term AZD8055 dosing used here does not immortalize human fibroblasts. Upon drug release, we find that AZD8055-exposed fibroblasts regain normal proliferation kinetics and reach senescence at the normal stage (Fig 7). Furthermore, we find that neither continuous AZD8055 exposure nor treatment followed by release results in any signs of either immortalization or transformation. It will be interesting to determine the effects of pulsed dosing, which may elicit beneficial senescence-reversal effects without triggering side effects such as activation of feedback loops that results from chronic mTORC inhibition [59]. Such pulsed therapies have already been reported in caloric restriction studies and most recently in use of a fasting-mimicking diet (FMD), where repeated short-term intervention led to long-term benefit [67]. Our findings overall suggest that inhibition of both mTORC1 and mTORC2 may be necessary to elicit maximal cellular changes necessary to reverse senescent phenotypes and that AZD8055 is a promising therapeutic candidate to reverse the detrimental effects of cellular senescence during aging.

## MATERIALS AND METHODS

Human neonatal foreskin fibroblast line HF043 (Dundee CELL products) was cultured in DMEM (Sigma) supplemented with 10% fetal calf serum (Gibco) in the absence of any added antibiotics, at 37°C in a humidified incubator with 5% CO_2_. Cells were monitored microscopically using an EVOS digital microscope (Life Technologies) and harvested when ∼80% confluent using TrypleExpress (Invitrogen). Following resuspension in DMEM with FCS, 20 μl of the cell suspension was counted and cell diameters measured using a Cellometer T4 (Nexelcom). Cells were seeded at 2×10^5^ per T25 flask (Greiner), or at 1×10^4^ in 24 well plates (Greiner). Population doublings (PD) were calculated as:

$$\text{PD}=\frac{\log_{10}(\text{total}\;\text{cells}\;\text{harvested}\;/\;\text{total}\;\text{cells}\;\text{seeded})}{\log_{10}2}$$

Cumulative population doublings (CPD) were calculated as the sum of PD values.

AZD8055 (Selleckchem) was reconstituted to 1mM in DMSO and stored in aliquots at −20°C protected from light. Prior to drug treatment, medium was removed, cells washed with PBS, then fresh medium supplemented with drug was added. Total volume of drug or DMSO added to the culture medium never exceeded 1:10,000 v/v. Doses of 35 nM and 70 nM were chosen as effective but non-toxic based on our preliminary studies (not shown).

### Fluorescence microscopy

To assess lysosomal content, cells were incubated with medium containing 50 nM Lysotracker red (Life Technologies) for 30 minutes at 37°C. Cells were imaged live using a Zoe microscope (BioRad). To account for differences in cell density arising from AZD8055 inhibition of cell proliferation, fluorescent signal (determined using Image J) was normalised against nuclear signal (DAPI). Mitochondrial content was assessed by adding 1μM Mitotracker Red (Molecular Probes) to cells in medium for 30 minutes at 37°C, washing twice with PBS and fixing with ice-cold methanol:acetone (1:1 v/v) for 10 min. Fixative was aspirated and cells washed with PBS prior to imaging. Stress fibers were detected by incubating for 40 min with 1μg/ml FITC-phalloidin (Sigma Aldrich) on cells that had been fixed with 3.7% formaldehyde (Sigma-Aldrich) in PBS for 5 minutes and washed twice with PBS prior to addition of FITC-phalloidin. Cells were then washed twice in PBS prior to imaging with either a BioRad Zoe or a Zeiss Axioskop II microscope. Images were quantified by Image J. Where appropriate, DNA was counterstained with NucBlue Live ReadyProbes Reagent (Life Technologies) (for live cell imaging) or ProLong® Gold Antifade Reagent with DAPI (Life Technologies) for fixed cells.

Scale bars for micrographs were determined using the appropriate microcopy software (BioRad SOFT-ZOE-Cell-Imager-UI-FW on Android operating system; Zeiss Axiovision SE64 Release 4.9.1 adjusted for Zeiss AxioCam Hr resolution), or by manual calculation from imaged rulers (Cellometer T4 and EVOS Core).

### SRB staining

Sulforhodamine B staining was performed as described in [36], but cells were photographed using an EVOS microscope (phase contrast), without dye solubilisation.

### SA-β-gal

Staining for (SA-β-gal) was performed using a Cell Signaling senescence-associated beta-galactosidase kit according to manufacturer's instructions.

### Immunoblotting

Cells were seeded at 2×10^5^ in T25 culture flasks (Greiner) and harvested by trypsinization as above, then pelleted by gentle centrifugation, washed in PBS and re-pelleted, then lysed in 30 μl RIPA buffer containing 1:100 Halt^TM^ protease and phosphatase inhibitors (Thermo Scientific). Lysates representing 2×10^4^ cells were heated for 5 minutes (95°C) in 1X NuPAGE LDS buffer (Novagen) containing 100 mM DTT prior to loading onto BioRad TGX 10% SDS-PAGE, blotted onto nitrocellulose (7 min 25V, 1.3A using a BioRad TurboBlot) then processed for immunoprobing as described previously [68], with the exception that primary antibodies were incubated with membrane at 1:500 dilution overnight at 4°C and secondary Ab (HRP-anti-rabbit or HRP-anti-mouse, both from Dako) were incubated at 1:1000 dilution for 30 mins at 37°C. Bound antibody was visualised by incubation with ECL solution for one minute at room temperature then exposure to HyperFilm MP (GE Healthcare) and development in an Xograph Compact 4 film processor. Anti-PCNA antibody was a polyclonal 3009 raised against the C terminus of PCNA [69]; all other primary antibodies used were purchased from Cell Signaling: anti mTOR phospho S2448 (D9C2) # 5536P; anti-mTOR (7C10) #2983A; anti-p70S6K-phospho T389 (108D2) #9234P; anti-p70 S6K #9202BC; anti-p27 #2552P; anti-phospho T37/46 4EBP-1 (236B4) #2855P. Note that all blots shown were of samples run on the same gel and the nitrocellulose filter was cut horizontally into sections prior to probing to avoid issues with non-equal loading or transfer. (Note that blots shown are representative of several replicates).

### Statistical analysis

Comparison of means between different populations (of different total cell number) used the student t test (one or two tailed according to the null hypothesis) where standard errors were similar. t was calculated according to

$$t^{2}=\frac{\left(\mu 1-\mu 2\right)}{\surd (\frac{1}{n1}+\frac{1}{n2})}$$

and t compared with values from t tables using degrees of freedom v = (n1 + n2 − 2).

## SUPPLEMENTARY FIGURE


