# Supplementary material for: Reversal of phenotypes of cellular senescence by pan-mTOR inhibition
Source: Aging (Albany NY). 2016 Feb 5;8(2):231–43. doi: 10.18632/aging.100872 (PMC4789579; doi:10.18632/aging.100872)
Supplement: Supplementary file 1 [file aging-08-231-s001.pdf]

SUPPLEMENTARY FIGURE

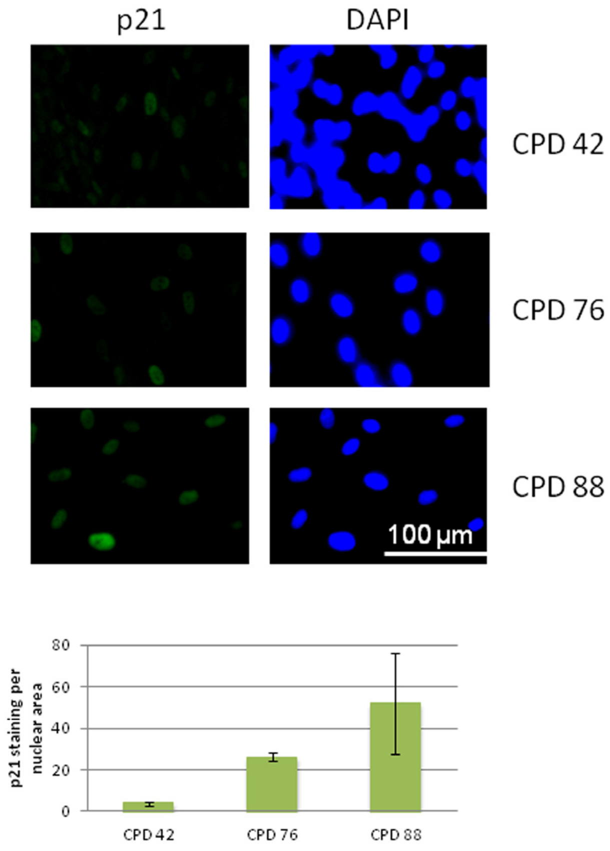

Supplementary Figure 1. Increase in senescent fraction shown by increase in p21 staining. HF043 fibroblasts were grown to CPD42, 76 or 88 and fixed prior to staining for p21. ImageJ quantification of p21 staining over nuclear area is shown in the graph (mean +/- standard deviation).
